# Proteomic Profiling Identifies Distinct Regulation of Proteins in Obese Diabetic Patients Treated with Metformin

**DOI:** 10.3390/ph16101345

**Published:** 2023-09-23

**Authors:** Awad Alshahrani, Ahmad Aljada, Afshan Masood, Muhammad Mujammami, Assim A. Alfadda, Mohthash Musambil, Ibrahim O. Alanazi, Mohammed Al Dubayee, Anas M. Abdel Rahman, Hicham Benabdelkamel

**Affiliations:** 1 Department of Medicine, Ministry of National Guard Health Affairs, Riyadh 11426, Saudi Arabia; shahranias@mngha.med.sa (A.A.); aldubayeemo@ngha.med.sa (M.A.D.); 2 King Abdullah International Medical Research Center, College of Medicine, King Saud bin Abdulaziz University for Health Sciences, Riyadh 11481, Saudi Arabia; 3 Department of Biochemistry and Molecular Medicine, College of Medicine, Al Faisal University, Riyadh 11533, Saudi Arabia; aaljada@alfaisal.edu; 4 Proteomics Resource Unit, Obesity Research Center, College of Medicine, King Saud University, P.O. Box 2925 (98), Riyadh 11461, Saudi Arabia; afsmassod@ksu.edu.sa (A.M.); aalfadda@ksu.edu.sa (A.A.A.); tmohthash@uliege.be (M.M.); 5 Endocrinology and Diabetes Unit, Department of Medicine, College of Medicine, King Saud University, Riyadh 11461, Saudi Arabia; mhmujammami@ksu.edu.sa; 6 University Diabetes Center, King Saud University Medical City, King Saud University, Riyadh 11461, Saudi Arabia; 7 Department of Medicine, College of Medicine and King Saud Medical City, King Saud University, P.O. Box 2925 (98), Riyadh 11461, Saudi Arabia; 8 Healthy Aging Research Institute, Health Sector, King Abdulaziz City for Science and Technology (KACST), P.O. Box 6086, Riyadh 11442, Saudi Arabia; ialenazi@kacst.edu.sa; 9 Metabolomics Section, Department of Clinical Genomics, Center for Genomics Medicine, King Faisal Specialist Hospital and Research Centre, Riyadh 11564, Saudi Arabia

**Keywords:** metformin, proteomics, obesity, type 2 diabetes mellitus, mass spectrometry

## Abstract

**Background**: Obesity and type 2 diabetes mellitus (T2DM) are characterized by underlying low-grade chronic inflammation. Metformin has been used as the first line of therapy in T2DM as it decreases hepatic glucose production and glucose intestinal absorption, enhances insulin sensitivity and weight loss, and is known to ameliorate inflammation. The mechanisms through which metformin exerts its effect remain unclear. Proteomics has emerged as a unique approach to explore the biological changes associated with diseases, including T2DM. It provides insight into the circulating biomarkers/mediators which could be utilized for disease screening, diagnosis, and prognosis. **Methods**: This study evaluated the proteomic changes in obese (Ob), obese diabetics (OD), and obese diabetic patients on metformin (ODM) using a 2D DIGE MALDI-TOF mass spectrometric approach. **Results**: Significant changes in sixteen plasma proteins (15 up and 1 down, ANOVA, *p* ≤ 0.05; fold change ≥ 1.5) were observed in the ODM group when compared to the Ob and OD groups. Bioinformatic network pathway analysis revealed that the majority of these altered plasma proteins are involved in distinct pathways involving acute-phase response, inflammation, and oxidative response and were centered around HNF4A, ERK, JNK, and insulin signaling pathways. **Conclusions:** Our study provides important information about the possible biomarkers altered by metformin treatment in obese patients with and without T2DM. These altered plasma proteins are involved in distinct pathways involving acute-phase response, inflammation, and oxidative response and were centered around HNF4A, ERK, JNK, and insulin signaling pathways. The presented proteomic profiling approach may help in identifying potential biomarkers/mediators affected by metformin treatment in T2DM and inform the understanding of metformin’s mechanisms of action.

## 1. Introduction

Obesity is recognized as a chronic relapsing condition contributing to increased morbidity and mortality, adding to the worsening global health burden. Globally, one in five adults is now known to be either overweight or obese [1]. An increasing prevalence of obesity is considered one of the main risk factors predisposing patients to the development of many non-communicable diseases, including T2DM, cardiovascular diseases, hypertension, coronary heart disease, and certain types of cancers. In concordance with the World Health Organization (WHO), overweight and obesity account for 44% of diabetes cases, 23% of ischemic heart disease cases, and around 7–41% of certain cancers [2].

Both obesity and T2DM are interrelated complex metabolic disorders with multifactorial pathophysiology. The close relationship between diabetes and obesity is highlighted by the term ‘diabesity’, reflecting that most individuals with diabetes are overweight or obese [3]. A characteristic feature of dysfunctional adipose tissue has been shown to secrete greater numbers of pro-inflammatory cytokines [4]. On the other hand, dysglycemia is associated strongly with a tendency to be overweight or obese (body mass index (BMI) > 30 kg/m^2^) [5]. Although BMI is used to characterize obesity, metabolic dysfunction is associated more with an increase in waist circumference or central abdominal adiposity. Moreover, obesity is a major causal factor in the development of hepatic insulin resistance. Chronic low-grade systemic inflammation has been proposed as an underlying mechanism that mediates insulin resistance, linking dysfunctional adipose tissue with the development of T2DM. One of the proposed underlying mechanisms points to the involvement of excess adiposity and the presence of free fatty acid (FFA) [6]. An increase in the levels of these markers results in a cascade of events leading to systemic chronic low-grade inflammation that originates from the inflamed adipose tissue, which in turn releases inflammatory mediators, recruits pro-inflammatory immune cells, and disrupts systemic metabolism and reduces insulin sensitivity [7].

Recent studies have reported that excess lipids in an obese state act as inflammatory signals, stimulating endoplasmic reticulum (ER) stress and inflammation in several cells and playing key roles in the development of insulin resistance [8]. The combined effects of obesity and diabetes are associated with an increased overall risk of premature death due to systemic complications resulting in cardiovascular disease, a primary complication and a leading cause of death [9]. Given the strong association between the two conditions, treatment modalities aim to use pharmacotherapies with a dual beneficial effect, resulting in lower blood glucose levels and supporting weight reduction.

Metformin, an insulin-sensitizing biguanide, has been used for decades in the treatment of hyperglycemia. The American Diabetes Association and seminal UK Prospective Diabetes Study (UKPDS) have suggested its use as the first-line therapy for treating T2DM and prediabetes, with patients randomized to this treatment group having reduced diabetes-related death rates and fewer hypoglycemic attacks than those treated with other pharmaceutical interventions [10]. Metformin modulates glucose and fatty acid metabolism, and improves glycemic control by reducing hepatic glucose production, enhancing peripheral insulin sensitivity, and blocking gastrointestinal glucose absorption [11,12]. At the molecular level, metformin acts by activating AMPK in various cell types in the body, including hepatocytes, skeletal muscle cells, endothelial cells, pancreatic beta cells, peripheral blood mononuclear cells, and platelets. Metformin has also been shown to have several beneficial effects in terms of cardiovascular risk factors. It is the only oral antihyperglycemic agent thus far associated with poorer macrovascular outcomes in patients with diabetes [13]. Besides the glucose-lowering effects, metformin use also causes weight loss through its actions on appetite regulatory pathways in the brain [14]. Additional influences on adipose- and gut-derived signals have been reported to exert beneficial effects with respect to aging [15]. It is also known to improve cardiovascular outcomes in patients with and without T2DM, which is attributed to increased vascular function and improved lipid profiles.

Proteomics aims to quantify and characterize all proteins that participate in the biological processes of an organism. Plasma proteome analysis can be used to identify diagnostic or prognostic biomarkers and provides insight into the mechanisms underlying disease development and progression. Proteomics, facilitated by recent advances in high-throughput technologies, has given us insight into the circulating biomarkers of both obesity and T2DM for screening, diagnosis, and prognosis of the disease. Although both obesity and diabetic profiles have been explored independently in earlier studies [16,17,18], to the best of our knowledge, there have been no proteomic studies examining the effect of metformin treatment on obese diabetic patients. On the other hand, metabolomic profiling in obese and diabetic patients with metformin treatment was evaluated in our previous study [19]. The present study explored the changes associated with metformin treatment using a 2D DIGE mass spectrometric approach and identified a few metabolic pathways associated with proteins through network pathway analysis.

## 2. Results

### 2.1. Clinical Characteristics and Demographics of the Study Population

The clinical features and demographic data of the study population are presented in Table 1. All patients were on daily doses of metformin ranging between 1000 and 2000 mg for at least 2 years.

### 2.2. Proteomic Analysis and Identification of Differentially Expressed Proteins

**Figure 1:**
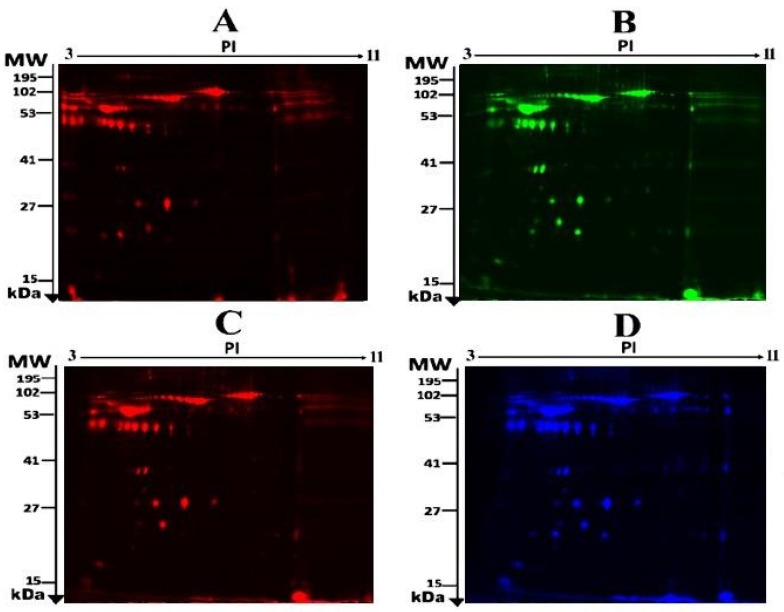
Representative fluorescent protein profile of a two-dimensional difference in gel electrophoresis (2D-DIGE) containing plasma sample from Ob samples labeled with Cy5 (**A**), OD samples labeled with Cy3 (**B**), ODM samples labeled with Cy5 (**C**), and pooled samples labeled with Cy2 (**D**).

**Figure 2:**
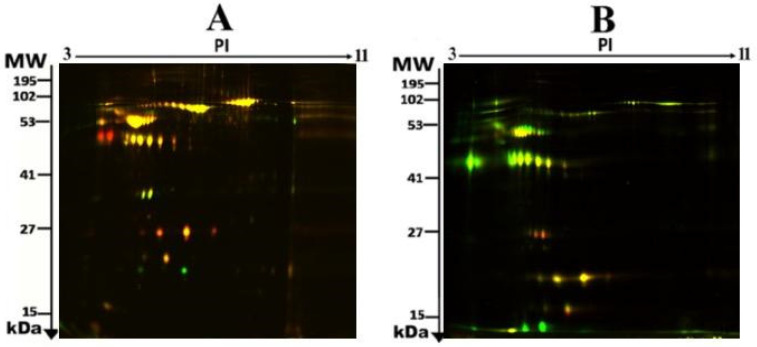
Representative fluorescent protein profiles of a two-dimensional difference in gel electrophoresis (2D-DIGE) containing merged samples from OD vs. Ob (**A**) and ODM vs. Ob (**B**).

**Figure 3:**
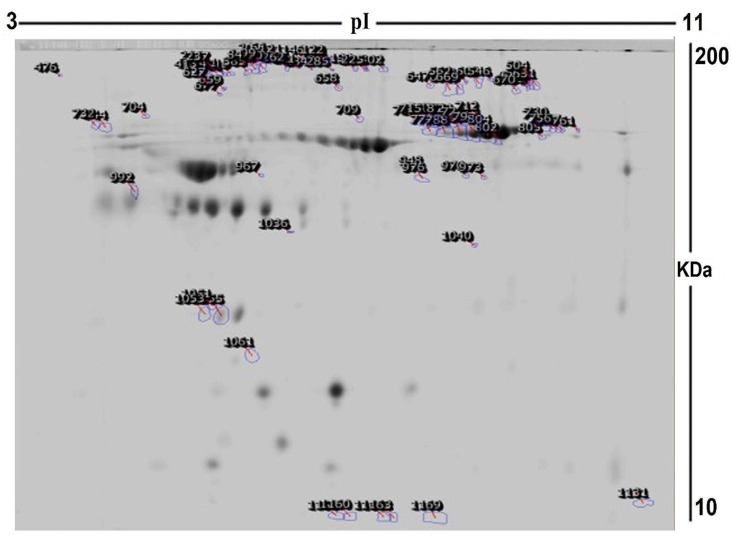
A representative preparatory 2D DIGE gel with numbered spots indicating proteins with differential abundance (defined as fold change ≥ 1.5, *p* ≤ 0.05) between Ob, OD, and ODM groups successfully identified with matrix-assisted laser desorption/ionization time of flight (MALDI TOF) mass spectrometry (MS) (MW, protein molecular weight; pI, isoelectric point).

To assess the differential protein expression among 10 Ob patients, 8 patients with OD, and 7 patients with ODM (25 samples from 13 gels), we performed 2D-DIGE and MALDI-TOF MS. Figure 1 shows the representative fluorescent protein profiles of a 2D-DIGE of an Ob sample labeled with Cy5 (A), an OD sample labeled with Cy3 (B), an ODM sample labeled with Cy5 (C), and a pooled sample labeled with Cy2 (D). Figure 2 shows 2D-DIGE containing merged samples from OD compared with Ob (A) and ODM compared with Ob (B). Figure 3 shows the 850 spots identified on the gels, among which 76 were significantly different (ANOVA, *p* ≤ 0.05; fold change ≥ 1.5) between the Ob, OD, and ODM groups. The spot patterns were reproducible across all 13 gels, leading to alignment and further analysis. Normalization across the complete set of gels and quantitative differential analysis of the protein levels were achieved using an internal standard with Cy2 labeling. The 76 spots showing a statistical significance between the three groups were then manually excised from the preparative gel for protein identification via MS.

Using MALDI-TOF mass spectrometry, peptide mass fingerprints (PMFs) were successfully employed to identify 30 out of the 76 protein spots excised from the preparative gel. Out of the 30 identified proteins matched to entries in the SWISS-PROT database by Mascot with high confidence scores, 24 spots were found to be unique protein sequences (Table 2 and Table S1). The sequence coverage of the proteins identified using PMFs ranged from 8% to 49%. In a few cases, variants of the same protein were found at several locations on the gel (Table 2, Figure 2).

A total of 30 proteins were identified, which could be used to compare the ODM and the cohort with and without T2DM. The comparison between ODM and Ob showed alterations between 24 proteins (20 protein spots were upregulated, 4 downregulated); between ODM and OD, 29 proteins were altered (27 protein spots were upregulated, 2 downregulated); and between OD and Ob, 14 proteins were altered (2 protein spots were upregulated, 12 downregulated) (Table 2, Figure 2).

A similar trend in the regulation was noted in 16 (12 unique) significantly differentially regulated proteins in the metformin-treated ODM group compared to the obese cohort (both OD and Ob groups). The significantly upregulated proteins between ODM and Ob included Apolipoprotein A-I (APOA1), ALB (albumin), serotransferrin (TRFE), hemopexin (HX), and ceruloplasmin (Ceru); the complete list is provided in Table 2. The only protein identified to have a decreased abundance in the ODM group compared to the OD and Ob groups was haptoglobin (HPT). Significant differences were also noted in seven proteins in the binary comparison between the ODM and OD groups, which were non-significant in the ODM vs. Ob groups. This comparison revealed an increase in six proteins, including calbindin 1 (CALB1), Hemoglobin subunit beta (HBB), Clathrin heavy-chain linker domain-containing protein 1 (CB063), and Activator of 90 kDa heat shock protein ATPase homolog 1 (AHSA1); the complete list is provided in Table 2. Only 1 protein Annexin A3 (ANXA3) increased in abundance in the ODM vs. Ob comparison. Interestingly, three proteins, namely alpha-2-macroglobulin (A2MG), PC4, and SFRS1-interacting protein (PSIP1), showed an increase and decrease trend, respectively, across the three binary comparisons.

Among the identified proteins, APOA1, TRFE, and ALBU were found in more than one spot on the gels, which could be associated with their post-translational modifications, cleavage by enzymes, or the presence of different protein species (Table 2).

### 2.3. Principal Component Analysis:

**Figure 4:**
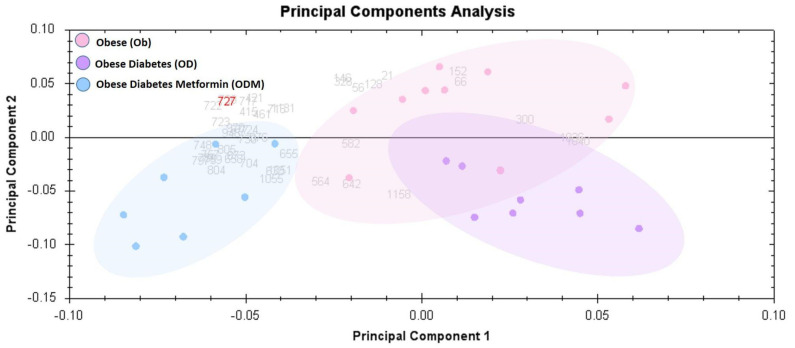
Principal component analysis (PCA) plot of the three first principal components. Altogether, they explained 68% of the selected spot’s variability. Colored dots and numbers are the representation of gels and spots, respectively.

The principal component analysis biplot was used as a graphical visualization to depict the separation of the three groups using the first two components, PC1 and PC2, only: ODM, obese and diabetes patients on metformin treatment; OD, obesity and diabetes; and OB, obese groups. Principal component analysis (PCA) carried out on all 30 spot features revealed that the three groups clustered distinctly from one another with a score of 68% (Figure 4). The data had a tendency to cluster into three groups with a clear and significant separation. Each dot in the biplot represents a patient, with each color representing different groups: obese (pink), obesity and diabetes (purple), and obese and diabetes patients on metformin (blue). The numbers represent significant proteins, according to ANOVA, with *p*-values lower than 0.05 and a fold change more than 1.5.

### 2.4. Network Pathway Analysis and Functional Classification of Proteins

Bioinformatic analysis using Ingenuity pathway analysis (IPA) was performed for all 30 of these differentially regulated proteins. To generate a protein–protein interaction network, the software computes a score based on the best fit obtained from the input data set of proteins and the biological function database. The generated network is favorably enriched for proteins with extensive and specific interactions. The interacting proteins are characterized as nodes and their biological relationships as a line. The canonical pathways are sorted down to decreasing log (*p*-value) of enrichment.

**Figure 5:**
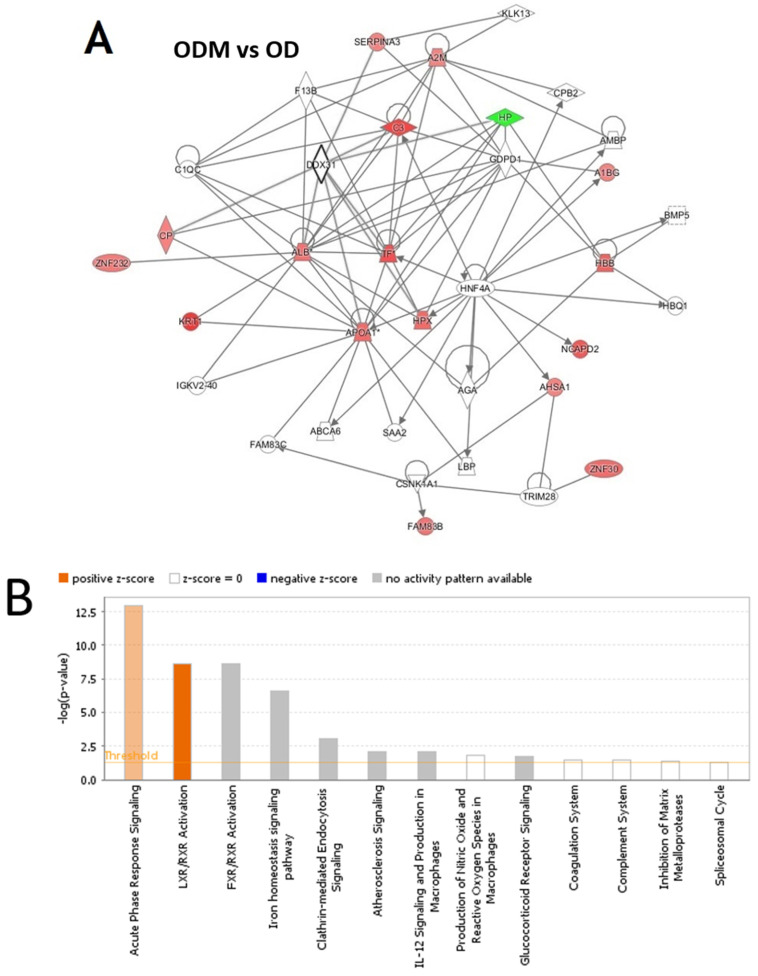

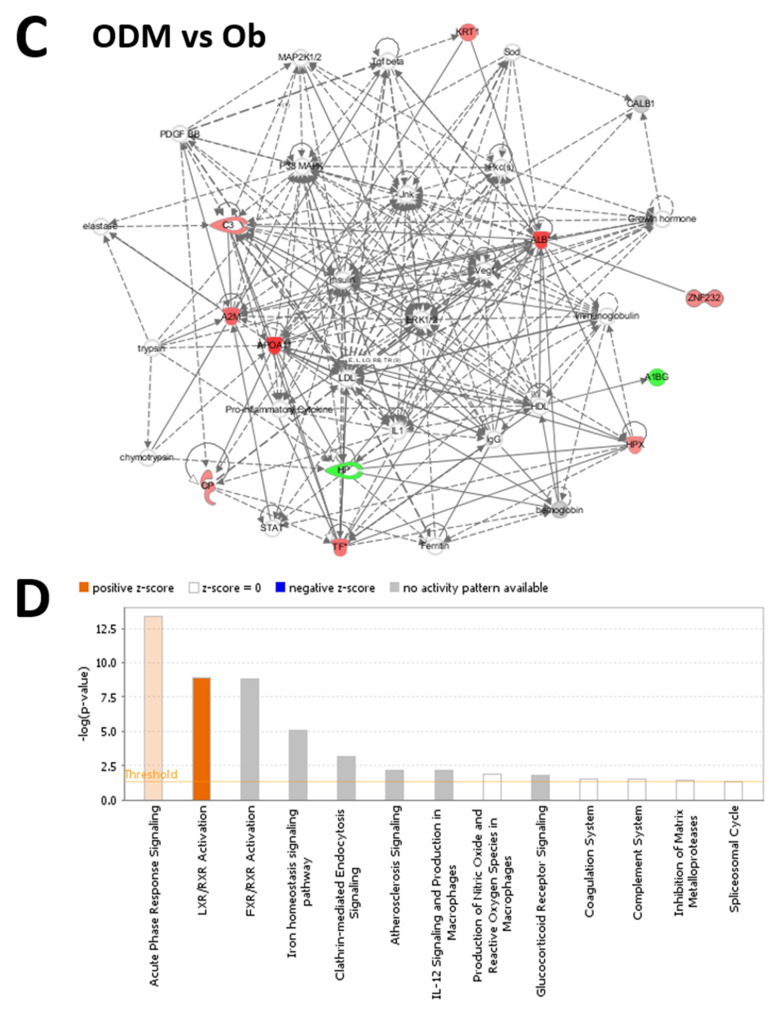
The most enriched interaction network of the differentially expressed proteins in ODM vs. OD (**A**,**B**) and ODM vs. Ob (**C**,**D**). Red nodes indicate upregulated proteins; green nodes indicate downregulated proteins. The central node of the pathway related to signaling of HNF4A (ODM vs. OD) and insulin, ERK1/2, JnK, and P38 MAPK (ODM vs. Ob) was found to be deregulated between the two states. Uncolored nodes were proposed by IPA and indicate potential targets that were functionally coordinated with the differentially expressed proteins. Solid lines indicate direct molecular interactions, and dashed lines represent indirect interactions. The diagram shows the top canonical pathways ranked by the *p*-values obtained by the IPA. The orange coloured bars indicate the predicted pathway activation based on the z-scores that are greater than or ezual. The higher the z-score the more darker is the colour. (**B**,**D** for ODM vs. OD and ODM vs. Ob, respectively).

Pathway analysis was carried out between the three group binary comparisons between ODM and OD, ODM and Ob, and OD and Ob. In the pathway analysis between ODM and OD, 17 proteins interacted directly or indirectly via protein networks (Figure 5A). Based on the data, 13 interaction networks were identified for the proteins exhibiting differential expression profiles (Figure 5B). The proposed highest interaction network pathway (score = 45) was related to cellular function and maintenance, neurological diseases, organismal injury, and abnormality signaling pathways. The five most interesting enriched canonical pathways included acute-phase response signaling (4.9% overlap, *p*-value: 1.09 × 10^−13^), LXR/RXR activation (4.9% overlap, *p*-value: 2.21 × 10^−9^), FXR/RXR activation (4.8% overlap, *p*-value: 2.56 × 10^−9^), the iron homeostasis signaling pathway (3.6% overlap, *p*-value: 2.56 × 10^−7^), and Clathrin-mediated endocytosis signaling (1.6% overlap, *p*-value: 9.61 × 10^−4^) (Figure S1).

The analysis between ODM and Ob revealed that 11 proteins interacted directly or indirectly via protein networks (Figure 5C). Based on the data, 13 interaction networks were identified for the proteins exhibiting differential expression profiles (Figure 5D). The highest scoring network (score = 31) incorporated 10 proteins (Figure S1). The proposed highest interaction network pathway was related to neurological diseases, organismal injury, and abnormality signaling pathways. The three most interesting enriched canonical pathways included acute-phase response signaling (4.9% overlap, *p*-value: 4.19 × 10^−14^), LXR/RXR activation (4.9% overlap, *p*-value: 1.24 × 10^−9^), FXR/RXR activation (4.8% overlap, *p*-value: 7.77 × 10^−6^), the iron homeostasis signaling pathway (2.9% overlap, *p*-value: 4.53 × 10^−9^), and Clathrin-mediated endocytosis signaling (1.6% overlap, *p*-value: 7.40 × 10^−4^) (Figure S1).

The analysis of the OD vs. Ob group revealed that six proteins interacted either directly or indirectly via protein networks (Figure S2A). Based on the data, nine interaction networks were identified for the proteins exhibiting differential expression profiles (Figure S2B). The proposed highest interaction network pathway (score = 29) was related to amino acid metabolism, energy production, post-translational modifications. The five most interesting enriched canonical pathways included acute-phase response signaling (3.2% overlap, *p*-value: 13.68 × 10^−9^), the iron homeostasis signaling pathway (2.9% overlap, *p*-value: 3.33 × 10^−6^), LXR/RXR activation (2.4% overlap, *p*-value: 1.07 × 10^−4^), FXR/RXR activation (2.4% overlap, *p*-value: 1.15 × 10^−4^), and Glucocorticoid receptor signaling (0.5% overlap, *p*-value: 9.21 × 10^−3^) (Figure S1).

**Figure 6:**
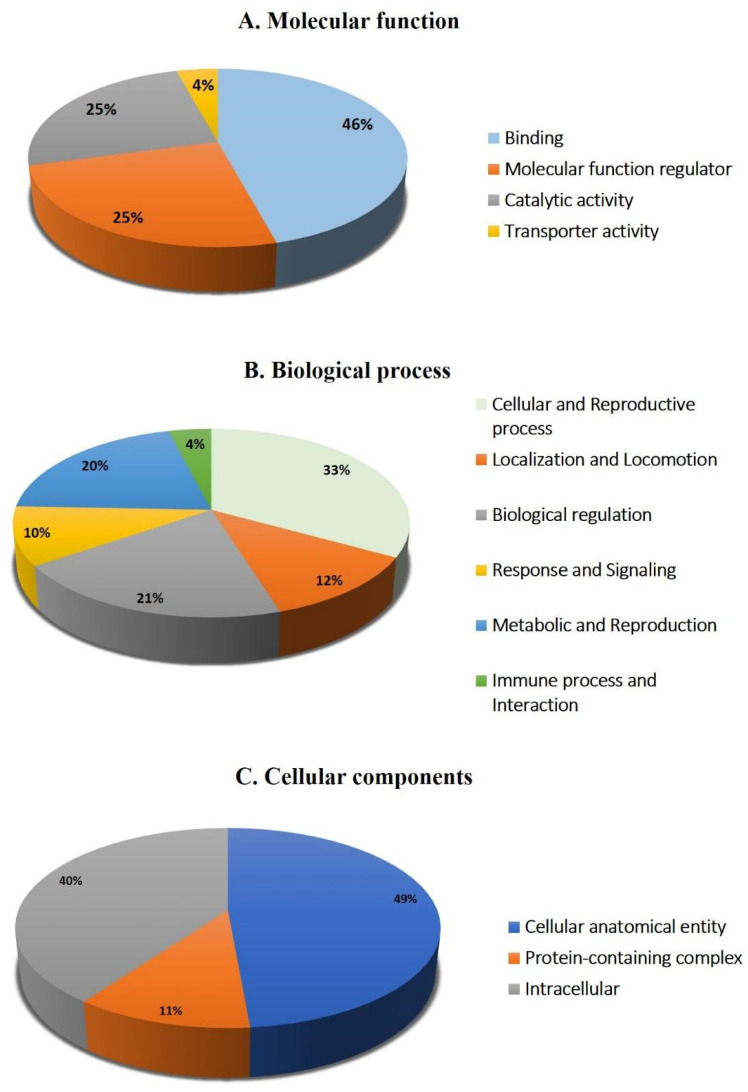
Comparative depiction (%) of identified proteins categorized into groups according to their molecular function (**A**), biological process (**B**), and cellular component (**C**).

The protein analysis through the evolutionary relationships (PANTHER) system was used for the classification of identified proteins according to their molecular function (Figure 6A), biological process (Figure 6B), and location (Figure 6C). The functional category showed that most of the differentially expressed proteins identified were enzymes with binding activity (46%), followed by molecular function regulators (25%) (Figure 6A). In regard to biological process, the majority of the identified proteins were involved in the cellular and reproductive process (33%), followed by biological regulation (21%) (Figure 6B). The majority of the identified proteins were located in a cellular, anatomical entity (49%), followed by the intracellular region (40%) (Figure 6C).

## 3. Discussion

In the present work, we compared the plasma proteomic profile in an obese cohort, divided into subgroups with and without diabetes (OD and Ob) and after metformin treatment (ODM). The 2D-DIGE MALDI/TOF-MS analysis conducted revealed significant differences with distinct clustering patterns in the identified proteins between the three groups. Among the 30 identified proteins in the data set, metformin treatment in the ODM group demonstrated a similar trend, with the abundance of 16 proteins (12 unique) in the obese cohort, with and without T2DM. The majority of these proteins are multifunctional proteins with varying roles in different metabolic processes. The major roles of these proteins in regulating the acute-phase response were identified. Regarding the regulation of oxidative stress (ALBU, APOA1, CERU, TRFE, HX, CO3, HPT), ALBU and APOA1 are well-known proteins involved in lipid metabolism, and TRFE, HX, and HPT are involved in iron metabolism and heme regulation.

Both obesity and diabetes are states of chronic inflammation with an underlying increase in inflammatory markers. Metformin is metabolized and acts mainly on the liver, which influences its overall metabolic activity. The liver is also the main site of the synthesis of acute-phase reactant proteins. In line with this finding, an increase in the number of acute-phase reactant proteins, ALBU, Apo-AI, CERU, TRFE, HX, and CO3, was noted post metformin administration. Besides mediating inflammation, these multifunctional proteins regulate several physiological processes, including lipid metabolism, act as scavenging proteins, and maintain the oxidative state. An increase in Apo A1 after metformin treatment suggests improved lipid mobilization and the success of reverse cholesterol treatment. APOA1 is the precursor for the high-density lipoproteins that have known anti-inflammatory and antioxidant functions that are impaired in T2DM and obesity [20]. Additionally, APOA1 is an anchor for other enzymes necessary for HDL maturation and is known to initiate reverse cholesterol transport. There is also evidence that APOA1 aids in antioxidative activity by removing or inhibiting the oxidation products of unsaturated fatty acids [21].

Metformin treatment increased the levels of CERU, TRFE, and HX in the ODM group. Obesity and diabetes are also associated with alterations in iron metabolism, mild inflammation, and oxidative stress [22,23]. Studies have provided inconclusive evidence for the changes in CRU and TRFE with diabetes, with a few studies showing a decrease while others demonstrated an increase [24,25]. An increase in the levels of CERU similar to that found in our study was noted by Chen et al. and Logie et al. [26] after metformin administration. CERU, TRFE, and HX, besides their anti-inflammatory actions, are also strong antioxidants and are differentially regulated in disturbances of copper and iron metabolism, respectively. An increase in the levels of these proteins has been shown to improve the redox state and prevent the harmful effects of oxidative stress. One of the major causes of increased oxidative stress in obesity and diabetes is the oxidation of the lipid moieties, reactive oxygen species generation by free fatty acids, and their continual accumulation in macrophages. This accumulation contributes to several biological disorders, including atherosclerosis, increased endoplasmic reticulum stress, and mitochondrial dysfunction, resulting in apoptosis [27,28]. An increase in the levels of CERU, TRFE, and HX points to the development of compensatory mechanisms that aim to increase antioxidant activity in patients treated with metformin. Metformin was also recently demonstrated to reduce oxidative stress in an obese and diabetic mouse model [29]. This finding was also supported by an associated decrease in HPT levels, a healthy antioxidant protein, in the metformin-treated group compared to the obese cohort. Polymorphisms in HP have been linked to complications arising from diabetes and obesity [30]. HP is an acute-phase protein that binds free hemoglobin and neutralizes oxidative damage. The levels of HPT and HX have been shown to have an inverse relationship wherein a decrease in HPT is balanced by an increase in the levels of HX to protect against the effects of ROS.

Besides its action on the liver, the anti-inflammatory effects of metformin have also been documented in multiple immune cells, including B cells, T cells, and macrophages [31,32]. An increase in the levels of Coro1A was noted in the ODM group compared to the obese cohort. Coro1A belongs to a family of actin-binding proteins that play a role in the rearrangement of the membrane cytoskeleton; this suggests that these proteins are critical due to their dynamic properties. They regulate actin-cytoskeleton-dependent processes such as cytokinesis, cell polarization, migration, phagocytosis, and trafficking in the leukocytes, along with aiding Ca signaling during the inflammatory process. Increased migration and infiltration of macrophages may occur in peripheral tissues, including pancreatic islets, liver, and adipose tissue, in response to inflammatory stimuli. An increase in the migratory capacity of macrophages, neutrophils, and lymphocytes has been shown for metformin. The functional implications of the other proteins identified to have an increased abundance after metformin treatment, including zinc finger proteins (69 and 232), FAM 83B, and K2C1, need to be further evaluated.

Interestingly, the levels of two significantly regulated proteins, A2MG and PSIP1, in the obese cohort did not show any changes with metformin treatment or T2DM. As the levels of these proteins were not significantly affected by the added comorbidity or the treatment, an alteration in their level can be considered a characteristic feature of obesity. A2MG showed an increase while PSIP1 decreased in abundance in the obese cohort. A2MG is a well-known global antiprotease carrier protein that binds to numerous growth factors and cytokines such as TNF-α, IL-6, and IL-1β. It is also known to be an anti-inflammatory protein identified to increase in obese individuals, similar to our finding [18]. In a recent animal study, A2MG was suggested as an acute or an early biomarker for those at risk of developing obesity or other conditions associated with obesity [33]. PSIP1 belongs to the hepatoma-derived growth factor (HDGF) family of proteins. Our findings are in line with Gómez-Ambrosi et al., who also observed a downregulation in the PSIP1 genes in obese omental tissue [34].

In the binary comparisons between the ODM, OD, and O groups, seven proteins showed significant differential regulation. Six proteins, including CALB and CB063, had an increased abundance, indicating that the increased abundance of these proteins was representative of the effect of T2DM on obese patients. The levels of CALB (also known as vitamin D-binding protein), a calcium-binding cytosolic protein, were increased in the ODM vs. OD group. Calbindin-D28k facilitates transcellular calcium diffusion, and a role for Calbindin has been established in modulating depolarization-stimulated insulin release from both isolated islets from calbindin-D28k KO mice and β-cell lines, suggesting that Calbindin can control the rate of insulin release [35].

In our study, only ANXA3 showed an increased abundance in the ODM vs. Ob groups, while its levels were not significant in the comparison of the ODM vs. OD groups. This indicates that metformin altered the levels of ANXA3 differentially between the OD and Ob groups. Annexin A3 (ANXA3), also known as Lipocortin III, is a soluble protein belonging to the Annexin superfamily of calcium-dependent phospholipid-binding proteins involved in regulating a diverse range of biological functions, including intracellular and extracellular signal transduction and interactions of cytoskeleton proteins, as well as anti-inflammation, anticoagulation, and angiogenesis [36]. Although dysregulation of ANXA1 and ANXA2 has been documented in diabetes [37,38,39], changes in ANXA3 have not been reported. ANXA3 promotes neutrophil granule aggregation in a calcium-dependent manner, regulates angiogenesis [40], and is a negative regulator of adipocyte differentiation [41].

Network pathway analysis of the ODM group compared to the OD and Ob groups showed the involvement of different metabolic pathways. The ODM vs. OD group showed that the dysregulated proteins centered around the regulation of HNF4A. HNF4α is known to directly regulate numerous genes encoding for proteins involved in glucose transport and glycolysis. At the same time, *HNF4A* polymorphisms are associated with defective insulin secretion, leading to an increased risk of type 2 diabetes mellitus and metabolic syndrome [42,43]. On the other hand, the metabolic networks associated with the dysregulated proteins in the ODM vs. Ob groups centered around regulating the pro-inflammatory ERK1, c-Jun N-terminal kinase (JNK), VEGF, p38 Kinase, and insulin signaling pathways. Previous studies have documented the established role of the inflammatory and stress response pathways in obesity and T2DM [8]. Metabolic stresses are known to activate several stress kinases, including the ERK1 and Jnk, which regulate the activity of insulin receptor substrate-1 [44]. The involvement of these proteins signifies the modulation of inflammation in patients with obesity and diabetes after metformin administration. The use of metformin in obese patients has the potential to decrease the inflammatory pathways. Its use in the obese can be recommended in clinics to all patients with obesity.

The strength of this study is that we compared the plasma proteomics of obese, obesity and diabetes, and obese and diabetes patients on metformin treatment. The selected patients did not have any comorbidities, and diabetes was treated with metformin only. The recent rapid advances in proteomic technologies have facilitated the analysis of protein signaling pathways in a high-throughput manner, which had greatly increased the understanding of mechanisms of action and accelerated biomarker discovery with drug (metformin) treatment, revolutionizing the landscape for disease treatment and diagnosis. This study will clearly play a key role in advancing personalized medicine with its associated benefits for the global community.

The study limitations include the design and limited sample size. Patient selection was also difficult, especially in the healthy obese group, as the majority of obese individuals are known to have impaired fasting glucose. Prospective studies with a larger cohort are needed to confirm the effects of metformin on protein signaling pathways in obese diabetes. We did not consider the effect of the dosage of metformin on protein signaling pathways. The 2D-DIGE mass spectrometry technique is both expensive and labor- and time-intensive, greatly increasing the cost and time needed to carry out the experiments.

## 4. Materials and Methods

### 4.1. Ethical Considerations and Informed Consent

All procedures performed in this study involving human participants followed the Declaration of Helsinki’s ethical standards and the International Conference on Harmonization–Good Clinical Practice (ICH-GCP) guidelines. This study was reviewed and approved by the Institutional Review Board (IRB) at King Saud University (approval number E-19-4234), the Institutional Review Board (IRB) at the King Faisal Specialist Hospital and Research Center (KFSHRC) (approval number 2170 013), Riyadh, Saudi Arabia, and the IRB of the Ministry of National Guard Health Affairs (MNGHA) (protocol RC12/105). Written informed consent was obtained from all participants.

### 4.2. Study Subjects

This study encompassed a cohort of Saudi participants, who were purposefully selected through a purposive sampling approach. All patients were assessed by their physicians during their clinic appointment, and informed consent was obtained. All the obese patients attending the clinic were recruited for this study. The patients were allowed to enroll even if they use statins, other cholesterol-lowering agents, angiotensin-converting enzyme inhibitors (ACE-Is), antihypertensive medications, non-steroidal anti-inflammatory drugs, or antioxidants, provided that the dosage remained stable for at least two months throughout the study. Obese subjects aged between 20 and 65 years of age, of any gender, were included if they demonstrated overall good health based on a comprehensive medical history and physical examination. A total of 25 samples (10 obese (Ob), 8 patients with obesity and diabetes (OD), and 7 obese and diabetes patients on metformin treatment (ODM)) were collected from patients referred to the Adult Diabetes Clinic at MNGHA. Anthropometric measurements were collected, and the BMI for each participant was calculated as body weight (in kilograms) divided by the square of body height (in meters). Patients who possessed a body mass index (BMI) within the range of 30 to 45 kg/m^2^ and exhibited normal fasting plasma glucose levels (≤6 mmoles/L) were included in the Ob group. OD incorporated subjects meeting similar age, gender, and health criteria, with a fasting glucose concentration of ≤13 mmoles/L and Hemoglobin A1c (HbA1c) levels under 10%. ODM patients on metformin were included if they met the age, gender, health, BMI, glucose, and HbA1c criteria and had been consistently taking a stable dose of metformin (1000–2000 mg) for at least two years.

From the cohort, patients were excluded from analysis in order to obtain a more homogenous population. For the obese diabetic patients, 5 patients had a smaller dose of 100–500 mg of metformin and 3 had a higher dose of 3000–4500 mg. These patients were excluded. In the ODM group, the patients who were on daily doses of metformin ranging between 1000 and 2000 mg for at least 2 years were included in the study. However, these subjects from the three groups were excluded from analysis as well to ensure that our analysis was based on a well-defined and consistent study population. *Blood samples were collected using venipuncture into EDTA-containing tubes (Vacutainer, BD Biosciences, San Jose, CA, USA) from each patient after an overnight fast. The plasma was separated* via *centrifugation (15 min, 3000× g) and was divided into several aliquots and stored at −80 °C for further analysis. The sample size was determined by conducting a power analysis using the Progenesis SameSpots non-linear dynamics statistical software (Version: v3.3, Nonlinear Dynamics Ltd., Newcastle, UK) to determine the minimum number of required biological replicates.*

### 4.3. Biochemical Analysis

Biochemical and hormone analyses were carried out using a Dimension Xpand Plus integrated clinical chemistry autoanalyzer (Siemens Healthcare Diagnostics, Molecules 2020, 25, 2831 13 of 18 Deerfield, IL, USA) [45]. HbA1c was analyzed using high-performance liquid chromatography and an ion-exchange chromatography assay (normal range 4.3–5.8%; Tosoh, Tokyo, Japan).

### 4.4. Sample Preparation and Protein Extraction

Plasma samples were thawed, and high-abundance plasma proteins (albumin, IgG) were depleted using Pierce^TM^ Top 12 Abundant Protein (Thermo Fisher Scientific, Waltham, MA, USA). Proteins were extracted using trichloracetic acid (TCA)/acetone precipitation, as described by Chen et al. [46]. Briefly, the depleted plasma samples were mixed (1:4 ratio) with ice-cold acetone containing 10% *w/v* TCA and vortexed for 15 s for uniform mixing. The mixture was incubated at 20 °C for 2 h for protein precipitation. After incubation, tubes were centrifuged at 1000× *g* for 15 min at 4 °C, and the pellet was solubilized in labeling buffer (7 M Urea, 2 M Thiourea, 30 mM Tris-HCl, 4% CHAPS, pH 8.5). The protein concentration of each sample was then determined in triplicate using the 2D-Quant Kit (GE Healthcare, Piscataway, NJ, USA).

### 4.5. Protein Labeling with Cyanine dyes

Equal amounts of protein (50 µg) from each sample from the OB, OD, and ODM groups were taken and labeled with 400 pmol of Cy3 and Cy5 dye. A mixture of an equal amount of all samples was then pooled, labeled with Cy2, and used as an internal standard; this standard was normalized and matched across gels to avoid gel-to-gel variation. A dye-switching strategy was applied during labeling to avoid dye-specific bias (Table S2), as previously described [18,19].

### 4.6. 2D-DIGE and Image Scanning

First-dimension analytical gel electrophoresis was performed, followed by second-dimension sodium dodecyl sulfate–polyacrylamide gel electrophoresis (SDS-PAGE) on 12.5% fixed concentration gels, as previously described [47,48]. The gels were scanned with a Sapphire Biomolecular Imager (Azure Biosystems, Dublin, OH, USA) and digitalized via the image analysis software Sapphire Capture system (Azure Biosystems, Dublin, OH, USA). Spot volumes were log-transformed to generate normally distributed data, and log-normalized volume instead of spot intensities was used in statistical processing to quantify differential expression. All spots were pre-filtered and manually checked before applying the statistical criteria (ANOVA test, *p* ≤ 0.05 and fold ≥ 1.5). Independent direct comparisons were made between the protein spots related to the OB, OD, and ODM groups, and the fold differences and *p*-values were calculated using one-way ANOVA. Spots that fulfilled the above-mentioned statistical criteria were submitted for further mass spectrometric (MS) analysis.

### 4.7. Colloidal Coomassie Blue Staining of the Preparative Gel

Total protein (1 mg) obtained from a pool of equal protein from the 25 plasma samples from both groups was separated via preparative two-dimensional (2D) gel electrophoresis. Gels were fixed in 40% (*v*/*v*) ethanol and 10% (*v*/*v*) acetic acid (overnight) and then washed (3×, 10 min each, ddH_2_O). The gels were incubated for 1 h in 34% (*v*/*v*) methanol containing 17% (*w*/*v*) ammonium sulphate and 3% (*v*/*v*) phosphoric acid) prior to the addition of 0.5 g/L Coomassie G-250. After 5–6 days, the stained gels were briefly rinsed with Milli-Q water and stored until the spots could be picked out and identified using MS [47,48,49].

### 4.8. Protein Digestion and MALDI Analysis

Coomassie-stained gel spots corresponding to the same spots that showed statistically significant differential abundance in the 2D-DIGE gels were excised manually. They were washed and digested according to previously described methods [47,48,49]. Finally, a mixture of tryptic peptides (0.8 μL) derived from each protein was spotted onto a MALDI target (384 MTP Anchorchip; 800 μm Anchorchip; Bruker Daltonics, Bremen, Germany). MALDI-MS (/MS) spectra were obtained using an Ultraflextreme time-of-flight (TOF) mass spectrometer equipped with a LIFT-MS/MS device (Bruker Daltonics) at reflector and detector voltages of 21 kV and 17 kV, respectively, as described previously [47,48]. PMFs were calibrated against a standard (peptide calibration standard II, Bruker Daltonics, Bremen, Germany). The PMFs were assessed using Flex Analysis software (version 2.4, Bruker Daltonics, Bremen, Germany)). MS data were interpreted using BioTools v3.2 (Bruker Daltonics). The peptide masses were searched against the Mascot search algorithm (v2.0.04, updated on 9 May 2020; Matrix Science Ltd., London, UK). The identified proteins were screened for a Mascot score of higher than 56 and *p* < 0.05.

### 4.9. Principal Component Analysis

The principal component analysis (PCA) was performed using Progenesis Same Spots software (Version: v3.3, Newcastle, UK) to determine and visualize the samples from the three groups, OD, ODM, and Ob. The PCA was performed on all the identified spots that exhibited statistically significant (ANOVA, *p* < 0.05) changes in abundance, as identified via MS.

### 4.10. Bioinformatics Analysis

Ingenuity pathway analysis (IPA), version 9.0 (Ingenuity Systems, Redwood City, CA, USA), was used to analyze protein interaction networks and the functions of the plasma proteins differentially expressed in the ODM group compared with the OB and OD groups. IPA software maps the UniProt IDs into the ingenuity knowledge base, the largest manually curated resource combining information from all published scientific studies. This software aids in determining the functions and pathways most strongly associated with the MS-generated protein list by overlaying the experimental expression data onto networks constructed from published interactions. The identified proteins were additionally classified into different categories according to their function and location using the PANTHER (protein analysis through evolutionary relationships) classification system (http://www.pantherdb.org, accessed on 23 January 2022).

### 4.11. Statistical Analysis

Data for the laboratory values are presented as means ± SD. The statistical significance of the difference between the three groups was analyzed with an unpaired Student’s *t*-test, and a value of *p*  <  0.05 was accepted as significant.

## 5. Conclusions

Our present study provides important information about the possible biomarkers altered by metformin treatment in obese patients with and without T2DM. Significant changes in sixteen plasma proteins were observed in the ODM group when compared to the Ob and OD groups. These altered plasma proteins are involved in distinct pathways involving acute-phase response, inflammation, and oxidative response and were centered around HNF4A, ERK, JNK, and insulin signaling pathways. The presented proteomic profiling approach may help in identifying potential biomarkers/mediators affected by metformin treatment in T2DM and inform the understanding of metformin’s mechanisms of action. Further studies using larger patient groups would be helpful to validate the role of these proteins in the mechanism of action of metformin.

## Figures and Tables

**Table 1:** The baseline clinical characteristics and demographic data of the study population.

	ODM (n = 7)	OD (n = 8)	OB (n = 10)
	Mean ± SD	Mean ± SD	Mean ± SD
Age (y)	48.71 ± 11.34	50 ± 9.78	40.4 ± 6.00 ‡
Gender (F/M)	6/1	1/7	9/1
BMI (kg/m^2^)	47.31 ± 6.88	30.74 ± 1.12 *	43.27 ± 6.78 ‡
Glucose (mM)	11.04 ± 3.57	12.2 ± 4.73	5.3 ± 0.43 *,‡
HbA1c (%)	9.19 ± 1.59	9.45 ± 3.09	5.75 ± 0.25 *,‡
Total Chol mmol/L	4.21 ± 0.92 †	5.78 ± 0.75	4.932 ± 0.70
LDL (mmol/L)	4.21 ± 0.92 ‡	5.78 ± 0.75 *	4.932 ± 0.70
HDL (mmol/L)	2.40 ± 0.67	3.7 ± 0.62	3.18 ± 0.76 †
Trig. (mM)	1.06 ± 0.27	0.96 ± 0.18	1.187 ± 0.15 †

ODM, obese and diabetes patients on metformin treatment; OD, obesity and diabetes; OB, obese; BMI, body mass index; HbA1c, glycated hemoglobin; HDL, high-density lipoprotein; LDL, low-density lipoprotein; statistically significant difference using ANOVA, * *p* ≤ 0.05. * Post hoc results are significantly different in comparison to ODM; † post hoc results are significantly different in comparison to OD; ‡ post hoc results are significantly different in comparison to OD.

**Table 2:** Identified proteins, with changes in abundance between obese diabetic metformin (ODM) vs. obese (Ob), ODM vs. obese diabetic (OD), and OD vs. Ob in plasma samples. Table 2 shows values for the average ratio between the two states, with their corresponding levels of fold changes and one-way ANOVA (*p*-value < 0.05) using 2D-DIGE (analysis type: MALDI-TOF; database: SwissProt).

S. No.	Protein Name	MASCOT ID ^a^	*p*-Value ^b^ (ANOVA)	Ratio ODM/Ob	Exp ^d^	Ratio ODM/OD ^c^	Exp ^d^	Ratio OD/Ob	Exp ^d^
1	Calbindin	CALB1_HUMAN	0.01	1	NS	2	UP	−1.89	DOWN
2	Apolipoprotein A–I	APOA1_HUMAN	0.01	2.6	UP	1.5	UP	-	NS
3	Albumin	ALBU_HUMAN	0.01	1.7	UP	1.5	UP	1.1	NS
4	Haptoglobin	HPT_HUMAN	0.01	−1.55	DOWN	−1.6	DOWN	1	NS
5	ANXA3_HUMAN	ANXA3_HUMAN	0.01	1.8	UP	1.1	NS	1.6	UP
6	Alpha-2-macroglobulin	A2MG_HUMAN	0.01	2.0	UP	1.5	UP	1.5	UP
7	Albumin	ALBU_HUMAN	0.02	2.4	UP	1.7	UP	1.4	NS
8	Hemopexin	HEMO_HUMAN	0.02	1.5	UP	1.8	UP	-	NS
9	Condensin complex subunit 1	CND1_HUMAN	0.02	1.7	UP	2	UP	-	NS
10	Pre-mRNA-splicing factor ISY1 homolog	ISY1_HUMAN	0.02	1.1	NS	1.7	UP	−1.5	DOWN
11	Hemoglobin subunit beta	HBB_HUMAN	0.02	-	NS	1.9	UP	−2.0	DOWN
12	Albumin	ALBU_HUMAN	0.02	1.6	UP	1.7	UP	-	NS
13	Complement C3	CO3_HUMAN	0.02	1.6	UP	2.2	UP	-	NS
14	Albumin	ALBU_HUMAN	0.03	1.5	UP	1.7	UP	-	NS
15	Serotransferrin	TRFE_HUMAN	0.03	1.5	UP	2	UP	−1.6	DOWN
16	Zinc finger protein 232	ZN232_HUMAN	0.03	1.5	UP	1.6	UP	-	NS
17	Serotransferrin	TRFE_HUMAN	0.04	1.5	UP	2.1	UP	−1.5	DOWN
18	Keratin, type II cytoskeletal 1	K2C1_HUMAN	0.04	1.7	UP	2.4	UP	−1.5	DOWN
19	Activator of 90 kDa heat shock protein ATPase homolog 1	AHSA1_HUMAN	0.04	-	NS	1.5	UP	−1.6	DOWN
20	Zinc finger protein 30	ZNF30_HUAMN	0.05	1.0	NS	1.8	UP	−1.7	DOWN
21	Serotransferrin	TRFE_HUMAN	0.05	1.8	UP	1.7	UP	1.1	NS
22	Alpha-1B-glycoprotein	A1BG_HUMAN	0.05	−1.5	DOWN	1.5	UP	−1.6	DOWN
23	PC4 and SFRS1-interacting protein	PSIP1_HUMAN	0.06	−1.5	DOWN	−1.5	DOWN	−1.5	DOWN
24	Clathrin heavy-chain linker domain-containing protein 1	CB063_HUMAN	0.05	1.2	NS	1.7	UP	−1.5	DOWN
25	Zinc finger protein 69	ZNF69_HUMAN	0.05	1.5	UP	1.6	UP	-	NS
26	Protein FAM83B	FA83B_HUMAN	0.05	1.6	UP	1.7	UP	1	NS
27	Ceruloplasmin	CERU_HUMAN	0.05	1.5	UP	1.5	UP	1	NS
28	Alpha-1-antichymotrypsin	AACT_HUMAN	0.05	−1.8	DOWN	1.5	UP	−2.1	DOWN
29	Apolipoprotein A-I	APOA1_HUMAN	0.05	1.8	UP	1.8	UP	-	NS
30	Coronin-1A	COR1A_HUMAN	0.05	1.6	UP	1.5	UP	1	NS

^a^ MASCOT id; ^b^ *p*-value (ANOVA); ^c^ ratio between the groups; ^d^ protein expression between the groups; NS: Non significant.

## Data Availability

The original contributions presented in the study are included in the article/Supplementary Material. Further inquiries can be directed to the corresponding authors.

## Supplementary Materials

The following supporting information can be downloaded at: https://www.mdpi.com/article/10.3390/ph16101345/s1, Figure S1: pathways and canonical pathways identified in the IPA functional analysis (A: ODM vs. Ob; B: ODM vs. OD; C: OD vs. Ob) Figure S2: the most enriched interaction network of the differentially expressed proteins in OD vs. Ob (S2A-B); Table S1: mass spectrometry list of significant differentially abundant proteins between obese (Ob), obese diabetic (OD), and obese diabetic metformin samples (ODM), using 2D-DIGE. Protein name, accession number, Mascot score, MS % coverage, protein MW, and pI values according to Uniprot database are listed. Table S2: dye-switching strategy applied during labeling to avoid dye-specific bias.
