# Supplementary material for: Proteomic Profiling Identifies Distinct Regulation of Proteins in Obese Diabetic Patients Treated with Metformin
Source: Pharmaceuticals (Basel). 2023 Sep 23;16(10):1345. doi: 10.3390/ph16101345 (PMC10609691; doi:10.3390/ph16101345)
Supplement: Supplementary file 1 [file pharmaceuticals-16-01345-s001.zip › pharmaceuticals-2542354-supplementary.pdf]

Supplementary data:

Figure S1: Pathways and canonical pathways identified in the IPA functional analysis (A: ODM vs Ob; B: ODM vs OD; C: OD vs Ob).

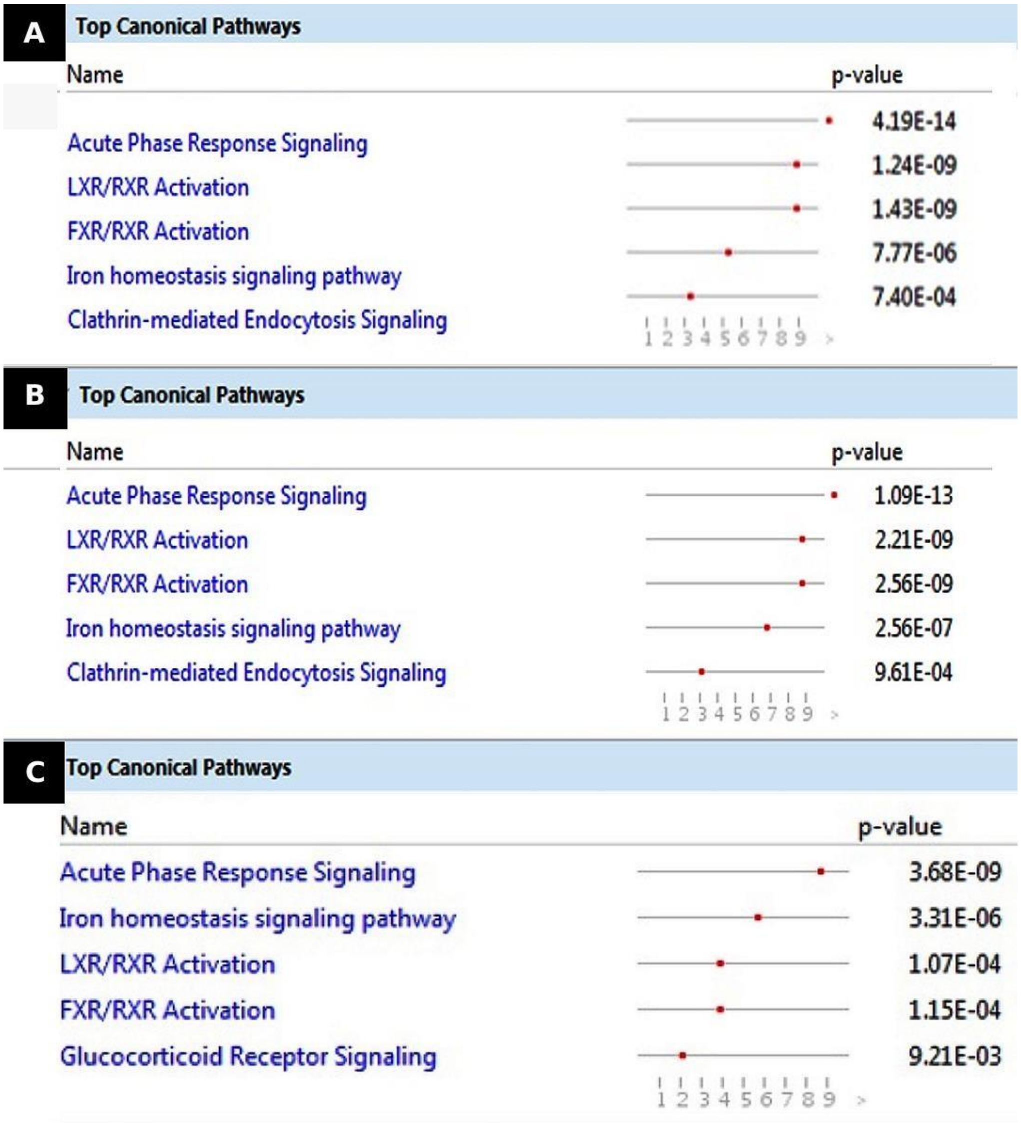

Figure S2: The most enriched interaction network of the differentially expressed proteins in OD vs Ob (S2A-B). Red nodes indicate up-regulated; green nodes indicate down-regulated. The central nodes of the pathway related to signaling of Insulin, ERK1/2, JnK, P38 MAPK, NFkB were found to be deregulated between the two states were found to be deregulated between the two states. Uncolored nodes are proposed by IPA and indicate potential targets that were functionally coordinated with the differentially expressed proteins. Solid lines indicate direct molecular interactions, and dashed lines represent indirect interactions (S2A). The diagram showing the top canonical pathways ranked by the P-values obtained by the IPA (S2B).

S2A

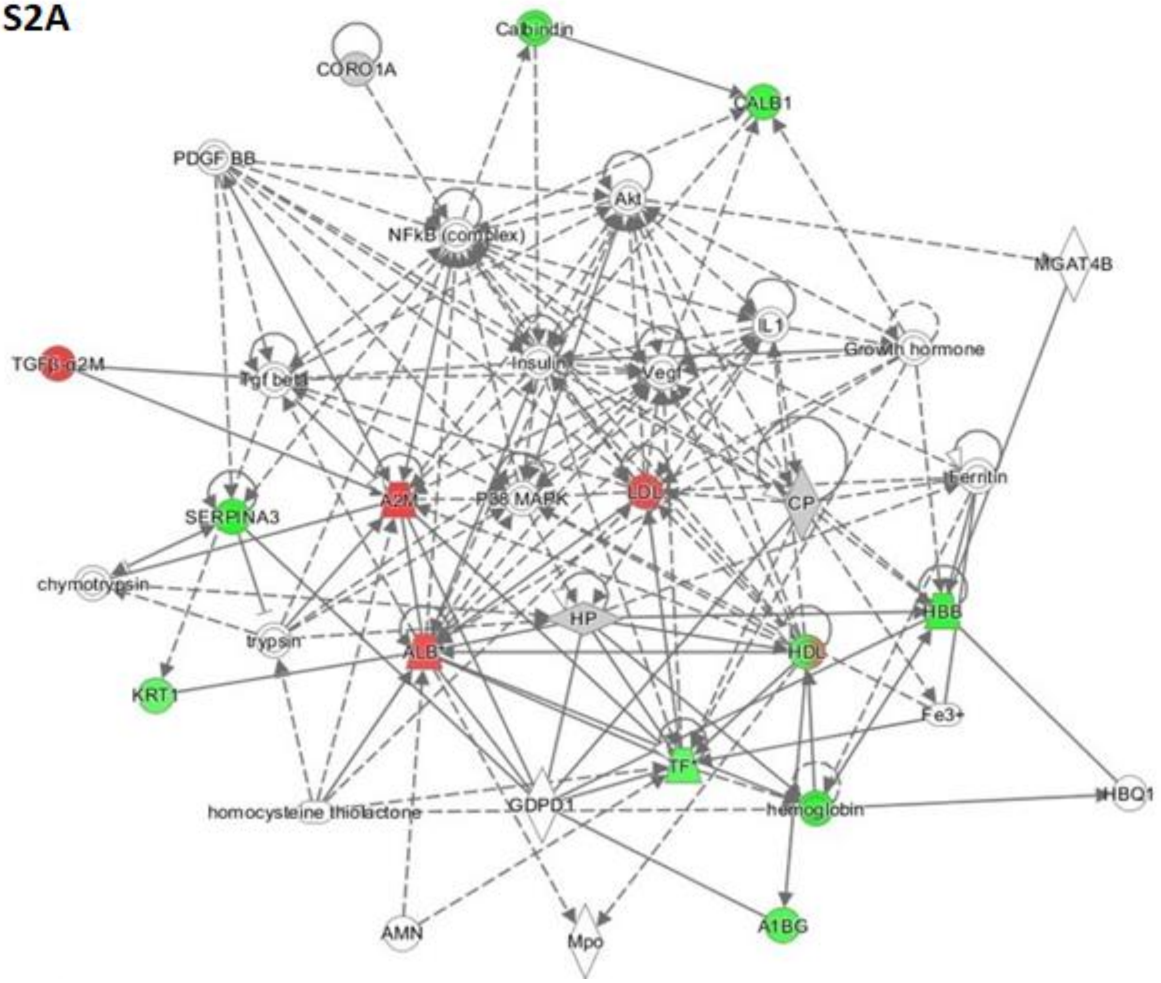

S2B

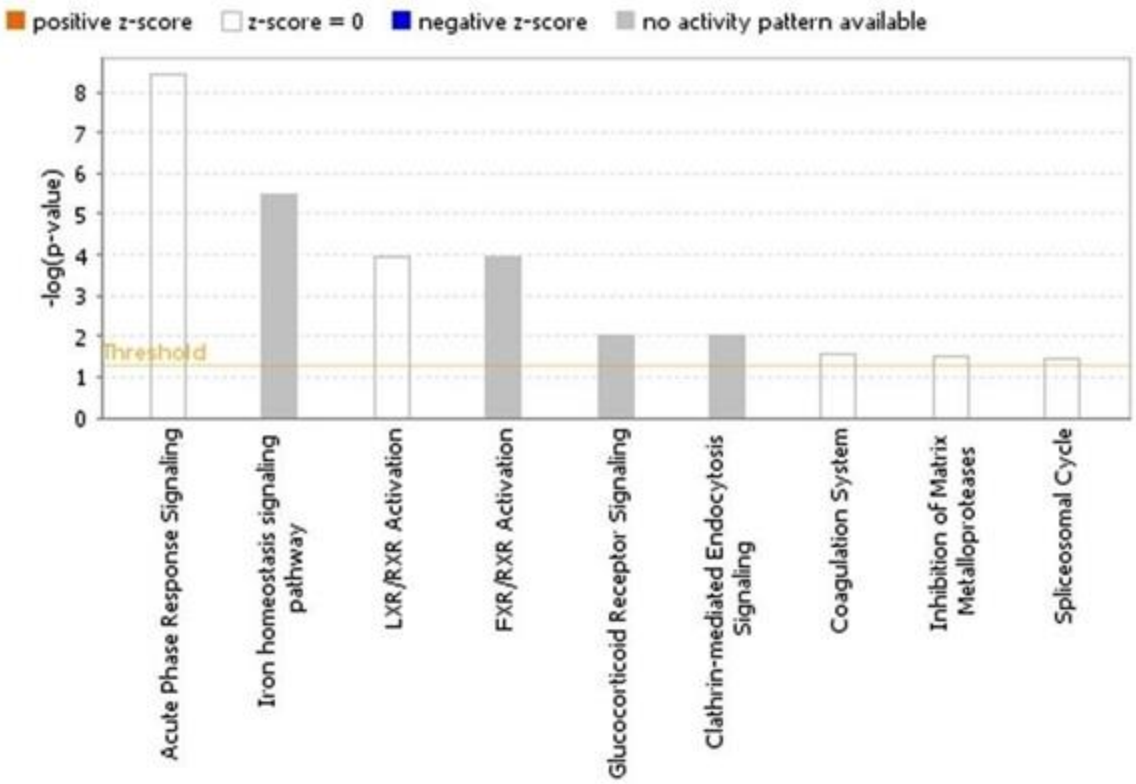

positive z-score
z-score = 0
negative z-score
no activity pattern available
-log(p-value)
8
7
6
5
4
3
2
1
0
Threshold
Acute Phase Response Signaling
Iron homeostasis signaling pathway
LXR/RXR Activation
FXR/RXR Activation
Glucocorticoid Receptor Signaling
Clathrin-mediated Endocytosis Signaling
Coagulation System
Inhibition of Matrix Metalloproteases
Spliceosomal Cycle

Table S1: Mass spectrometry list of significant differentially abundant proteins between Obese (Ob), Obese diabetic (OD),and Obese Diabetic Metformin samples (ODM), using 2D-DIGE with. Protein name, accession number, Mascot score, MS % coverage,protein MW and pI values according to Uniprot database are listed.

Sl no:	Spot No[a]	Accession No[b]	Protein Name	MASCOT ID	Pi[c]	MW[d]	Cov%	Score[e]
1	727	P05937	Calbindin	CALB1_HUMAN	4.70	30291	19	98
2	1055	P02647	Apolipoprotein A-I	APOA1_HUMAN	5.56	30759	41	96
3	673	P02768	Albumin	ALBU_HUMAN	5.92	71317	24	90
4	1036	P00738	Haptoglobin	HPT_HUMAN	6.13	45861	23	74
5	642	P12429	ANXA3_HUMAN	ANXA3_HUMAN	5.63	36524	22	58
6	630	P01023	Alpha-2-macroglobulin	A2MG_HUMAN	6.00	164614	15	110
7	658	P02768	Albumin	ALBU_HUMAN	5.92	71317	22	69
8	789	P02790	Hemopexin	HEMO_HUMAN	6.55	52385	20	57
9	805	Q15021	Condensin complex subunit 1	CND1_HUMAN	6.19	158508	9	66
10	723	Q9ULR0	Pre-mRNA-splicing factor ISY1 homolog	ISY1_HUMAN	5.69	37771	19	68
11	1131	P68871	Hemoglobin subunit beta	HBB_HUMAN	6.75	16102	49	78
12	655	P02768	Albumin	ALBU_HUMAN	5.92	71317	15	64
13	759	P01024	Complement C3	CO3_HUMAN	6.02	188569	13	77
14	797	P02768	Albumin	ALBU_HUMAN	5.92	71317	41	90
15	748	P02787	Serotransferrin	TRFE_HUMAN	6.81	79280	29	109
16	943	Q9UNY5	Zinc finger protein 232	ZN232_HUMAN	6.28	48456	24	65
17	757	P02787	Serotransferrin	TRFE_HUMAN	6.81	79280	37	165
18	461	P04264	Keratin, type II cytoskeletal 1	K2C1_HUMAN	8.15	66170	16	68
19	21	O95433	Activator of 90 kDa heat shock protein ATPase homolog 1	AHSA1_HUMAN	5.41	38421	15	57
20	992	P17039	Zinc finger protein 30	ZNF30_HUAMN	9.33	64035	17	67
21	631	P02787	Serotransferrin	TRFE_HUMAN	6.81	79280	29	127
22	659	P04217	Alpha-1B-glycoprotein	A1BG_HUMAN	5.58	54809	21	66
23	192	O75475	PC4 and SFRS1-interacting protein	PSIP1_HUMAN	9.15	60181	14	93
24	434	Q8NHS4	Clathrin heavy chain linker domain-containing protein 1	CB063_HUMAN	6.09	68247	13	88
25	670	Q9UC07	Zinc finger protein 69	ZNF69_HUMAN	9.13	67999	16	72
26	709	Q5T0W9	Protein FAM83B	FA83B_HUMAN	9.04	115185	8	87
27	669	P00450	Ceruloplasmin	CERU_HUMAN	5.44	122983	9	77

28	732	P01011	Alpha-1-antichymotrypsin	AACT_HUMAN	5.33	47792	16	67
29	1053	P02647	Apolipoprotein A-I	APOA1_HUMAN	5.56	30759	20	67
30	546	P31146	Coronin-1A	COR1A_HUMAN	6.25	51678	8	60

[a] Spot number.

[b] Protein accession number for SWISSPROT Database.

[c] Theoretical isoelectric point.

[d] Theoretical relative mass.

[e] MASCOT score.

Table S2: Dye-switching strategy applied during labeling to avoid dye-specific bias. A total of 26 samples were run on 13 2D-PAGE gels. Samples were labeled randomly with Cy3 and Cy5, and a pooled sample was used as an internal standard and was stained with Cy2 (Ob:Obese, OD: Obese diabetic and ODM: Obese diabetic metformin treated).

Gel	Cy3	Cy5	Cy2
1	Ob1	OD1	Pool
2	ODM1	Ob2	Pool
3	OD2	Ob3	Pool
4	OD3	ODM2	Pool
5	Ob4	OD4	Pool
6	OD5	ODM3	Pool
7	ODM4	O5	Pool
8	O6	-	Pool
9	Ob7	ODM5	Pool
10	ODM6	Ob8	Pool
11	Ob9	OD7	Pool
12	OD6	Ob10	Pool
13	OD8	ODM7	Pool
